# Presence of *Leishmania* sp. amastigotes in the reproductive tract of dogs with visceral leishmaniasis

**DOI:** 10.1590/0037-8682-0237-2023

**Published:** 2024-09-20

**Authors:** Marla de Oliveira D’Esquivel, Vanessa Paulino da Cruz Veira, Daniel Moreira de Avelar, Érika Monteiro Michalsky, Nathália Cristina Lima Pereira, Consuelo Latorre Fortes-Dias, Edelberto Santos Dias

**Affiliations:** 1 Prefeitura Municipal de Teófilo Otoni, Teófilo Otoni, MG, Brasil.; 2 Instituto Federal do Norte de Minas, Campus Salina, Salinas, MG, Brasil.; 3 Instituto René Rachou, Fundação Oswaldo Cruz, Belo Horizonte, MG, Brasil.; 4 Fundação Ezequiel Dias, Diretoria de Pesquisa e Desenvolvimento, Belo Horizonte, MG, Brasil.

**Keywords:** Canine visceral leishmaniasis, Canine reproductive tract, Venereal transmission, Leishmania

## Abstract

**Background:**

Although canine visceral leishmaniasis (CVL) transmission primarily occurs through the bite of phlebotomine sand flies infected with *Leishmania infantum*, alternative routes may exist.

**Methods:**

Thirty-four dogs diagnosed with CVL were sampled for parasitological investigation in tissues from the reproductive tract.

**Results:**

Amastigotes of *Leishmania* sp. were present in 79% (27/34) of the reproductive system samples, with distinct infection rates depending on the tissue.

**Conclusions:**

Our data confirms that alternative routes, such as horizontal and vertical transmissions, should be considered in the epidemiological chain of CVL.

Leishmaniasis is a zoonotic disease caused by a protozoa of the *Leishmania* genus (Kinetoplastida:Trypanosomatidae), and is transmitted by phlebotomine sand flies (Diptera:Psychodidae:Phlebotomine). *Leishmania infantum* Nicolle 1908 is the etiological agent of leishmaniasis in the Americas, the Middle East, and some Central Asian countries^1^. Domestic dogs (*Canis lupus familaris*) are considered the main reservoirs of *Le. infantum*, which play a fundamental role in canine visceral leishmaniasis (CVL) transmission to humans. In Brazil, *Lutzomyia longipalpis* (Lutz & Neiva,1912) is the main *Leishmania* vector^2^. 

Among insect vectors, *Leishmania* spp. are present in flagellate or promastigote forms. During blood meals, female phlebotomine sand flies previously infected with *Leishmania* inoculate metacyclic promastigotes into vertebrate hosts. Promastigotes are phagocytosed by dermal macrophages and differentiate into amastigotes within phagocytic cells. This is followed by intense reproduction of amastigotes by binary division until the cell ruptures and parasites are released. Free amastigotes are phagocytosed by new macrophages in a continuous process, leading to hematogenous and lymphatic dissemination to other tissues rich in mononuclear phagocytic cells, such as the lymph nodes, liver, spleen, and bone marrow^3^. *Leishmania* parasites exist as amastigotes in vertebrate hosts.

Until two decades ago, CVL transmission was restricted to the bites of the infected vectors. However, other possible routes of infection have been described, such as venereal and vertical transmission^4^ ^-^ ^6^, in places without insect vectors. However, the contribution of these alternative routes to the epidemiology of CVL in areas endemic to phlebotomine sand fly vectors remains unknown.

In this cross-sectional descriptive study, we investigated the presence of *Leishmania* sp. amastigotes in the reproductive tract of CVL-positive dogs from an endemic area in Brazil. Our aim was to contribute to the knowledge on other possible routes of CVL transmission.

The study was developed between November 2020 and September 2021 in the municipality of Teófilo Otoni (17°51’28"Sul 41°30’18"W) in the Northeast of the Brazilian state of Minas Gerais. This region is endemic for CVL, with 1,064 confirmed cases reported from 2017 to 2021. 

Our samples included canine males and females of different breeds and ages taken by owners for CVL testing at the Zoonosis Center of Teófilo Otoni (Minas Gerais State, Brazil) or stray dogs collected by health agents for the same purpose. Blood samples were collected via venous puncture of the auricular marginal vein using disposable microlancets and tested using a rapid dual-path platform for CVL (TR-DPP^®^-LVC, Bio-Manguinhos, Rio de Janeiro, Brazil). If positive, 2-3 mL of blood was collected via the cephalic vein using a disposable syringe with a 25 × 7-mm needle for diagnosis confirmation. The samples were further tested using an enzyme-linked immunosorbent assay (ELISA; EIE LVC, Bio-Manguinhos, Rio de Janeiro, Brazil) in an accredited laboratory (Fundação Ezequiel Dias, Minas Gerais). Dogs with positive results in both tests were considered seropositive for CVL and were recommended for euthanasia, according to the protocol of the Program for Surveillance and Control of Visceral Leishmaniasis of the Brazilian Ministry of Health. 

Sample I consisted of 34 dogs (17 males and 17 females, aged 2-7 years). Notably, 91% (31/34) of the participants were mongrels. The inclusion criteria for sample I were seropositivity for TR-DPP and ELISA results, and euthanasia recommendations. The selected dogs were examined by veterinary physicians and classified as asymptomatic or symptomatic according to the absence or presence of at least one clinical sign suggestive of VL (i.e., cutaneous alterations, such as depilation, dermatitis, and ulcers; onychogryphosis; keratoconjunctivitis; loss of weight; emaciation; and rigidity of posterior limbs). Before euthanasia, the animals were anesthetized intravenously with 100 mg/mL ketamine hydrochloride at a dose of 2-5 mg/kg. After sedation, 2-5 mg/kg of 10% potassium chloride was administered. Tissue fragments were collected from the testicles, epididymis (head, body, and tail), prostate, ovaries, uterine body, vagina, and spleen. Sample collection and euthanasia were performed individually, with no contact between the animals.

Sample II comprised asymptomatic TR-DPP-negative dogs transported to the Mobile Castration Unit of the Municipal Program of Population Control. The animals were manually restrained by their owners and were administered with 0.2% acepromazine intramuscularly at a concentration of 0.025-0.1 mL/kg. After sedation, the animals were locally anesthetized with 0.25-0.75 mg/kg lidocaine for the castration procedure. Testicular, epididymal, prostate, and spleen fragments were collected and imprinted onto microscope slides. The animals rested in the bays until they recovered fully and returned home. 

Imprints of the tissues collected *ante* and *post mortem* were prepared on microscopic slides and stained panoptically. The presence of *Leishmania* sp. amastigotes was investigated by examining 200 fields in immersion oil under an optical microscope equipped with a 100 × objective lens. The absence of amastigotes in all fields was considered negative^7^. 

This study was conducted in accordance with the ethical principles of animal experimentation adopted by the Brazilian College of Animal Experimentation, and approved by the Ethical Committee on the Use of Animals of the Instituto Federal do Norte de Minas Gerais (Protocol no. 015/2020). Euthanasia was performed according to the technical norms defined in Resolution No. 1000 of the Federal Council of Veterinary Medicine, dated 11/05/2012. Before the procedure, dog owners, if available, signed euthanasia agreements. The collection of tissue samples was authorized by the owners by signing the Free and Informed Consent Form and the Institutional Consent Form of the Municipal Health Department of Teófilo Otoni.

A total of 894 dogs were tested for CVL. Among the 114 positive dogs, 46% (52/114) were male and 54% (62/114) were female. Among the 34 dogs in sample I, 31 showed at least one clinical sign of CVL: weight loss and cutaneous lesions (non-pruritic exfoliative dermatitis with or without alopecia, erosive/ulcerative dermatitis, nodular dermatitis, papular dermatitis, and onychogryphosis), mucous and mucocutaneous lesions (oral, genital, and nasal), and eyepieces (blepharitis, conjunctivitis, common or dry keratoconjunctivitis, anterior uveitis, and endophthalmitis).

Distinct tissue fragments were necropsied from seropositive males and females. Imprint analysis of canine tissues from sample I revealed the presence of *Leishmania* sp. amastigotes in 27 tissues (16/17 males [94%] and 11/17 females [65%]). Considering tissues from the reproductive tract, the percentages decreased to 88% and 35% in males and females, respectively. Clinical signs of CVL were present in 76% of the males and 35% of the females with *Leishmania* parasites in the imprints (Figure 1).

**FIGURE 1:**
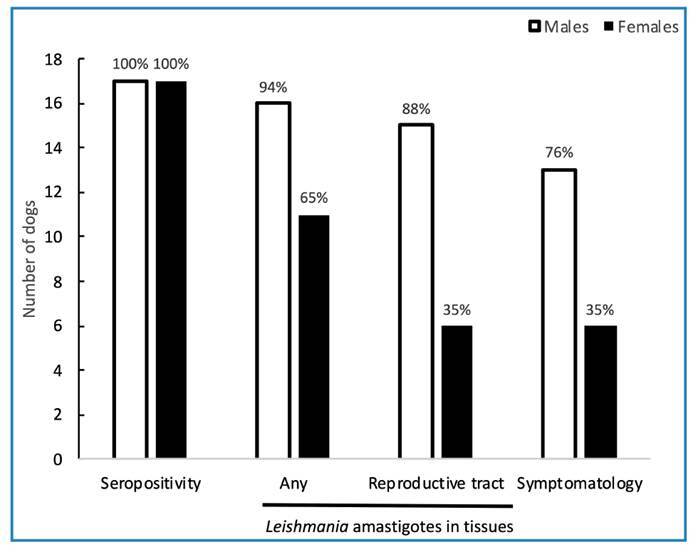
Characteristics of the canine sample I (n=34). Seropositivity, detection of *Leishmania* amastigotes in tissue imprints, and presence of clinical signs of CVL. CVL, canine visceral leishmaniasis.

*Leishmania* amastigotes were unequally distributed in the tissues of the male and female canine reproductive tracts (Figure 2). *Leishmania* sp. amastigotes were detected in the imprints of canine vaginal tissue, but not in any of the tissues examined from Sample II (TR-DPP-negative dogs).

**FIGURE 2:**
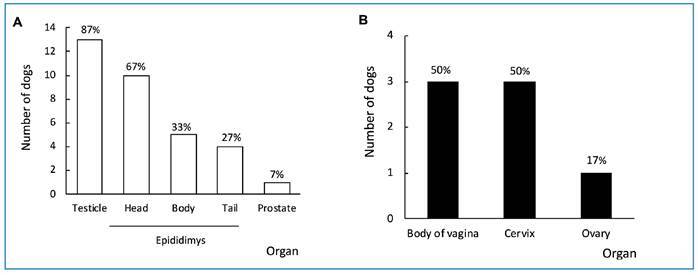
Detection of *Leishmania* sp. amastigotes in tissues of the reproductive tract of dogs seropositive for VL from sample I. **A.** Males (n=15); **B.** Females (n=6). **VL:** visceral leishmaniasis.

The limitations of the present study include the absence of histopathological or immunohistochemical evidence of *Leishmania* amastigotes in the infected tissues and the lack of specific identification of the infecting parasite. Attempts to extract DNA from the imprints were unsuccessful, possibly because of an insufficient number of parasites in the tissues. However, our findings open a new window for exploring alternative routes in the epidemiological chain of CVL transmission in areas endemic to the disease.
